# Assessment of New Onset Arrhythmias After Transcatheter Aortic Valve Implantation Using an Implantable Cardiac Monitor

**DOI:** 10.3389/fcvm.2022.876546

**Published:** 2022-05-16

**Authors:** Nikolas Nozica, George C. M. Siontis, Elena Georgieva Elchinova, Eleni Goulouti, Masahiko Asami, Joanna Bartkowiak, Samuel Baldinger, Helge Servatius, Jens Seiler, Hildegard Tanner, Fabian Noti, Andreas Haeberlin, Mattia Branca, Jonas Lanz, Stefan Stortecky, Thomas Pilgrim, Stephan Windecker, Tobias Reichlin, Fabien Praz, Laurent Roten

**Affiliations:** ^1^Department of Cardiology, Inselspital, Bern University Hospital, University of Bern, Bern, Switzerland; ^2^Division of Cardiology, Mitsui Memorial Hospital, Tokyo, Japan; ^3^Clinical Trials Unit, University of Bern, Bern, Switzerland

**Keywords:** TAVI, implantable cardiac monitor (ICM), pacemaker (PM), atrial fibrillation, bundle branch block (BBB), AV block, ventricular tachycardia (VT)

## Abstract

**Background:**

Transcatheter aortic valve implantation (TAVI) is associated with new onset brady- and tachyarrhythmias which may impact clinical outcome.

**Aims:**

To investigate the true incidence of new onset arrhythmias within 12 months after TAVI using an implantable cardiac monitor (ICM).

**Methods:**

One hundred patients undergoing TAVI received an ICM within 3 months before or up to 5 days after TAVI. Patients were followed-up for 12 months after discharge from TAVI for the occurrence of atrial fibrillation (AF), bradycardia (≤30 bpm), advanced atrioventricular (AV) block, sustained ventricular and supraventricular tachycardia.

**Results:**

A previously undiagnosed arrhythmia was observed in 31 patients (31%) and comprised AF in 19 patients (19%), advanced AV block in 3 patients (3%), and sustained supraventricular and ventricular tachycardia in 10 (10%) and 2 patients (2%), respectively. Three patients had a clinical diagnosis of sick-sinus-syndrome. A permanent pacemaker (PPM) was implanted in six patients (6%). The prevalence of pre-existing AF was 28%, and 47% of the patients had AF at the end of the study period. AF burden was significantly higher in patients with pre-existing [26.7% (IQR 0.3%; 100%)] compared to patients with new-onset AF [0.0% (IQR 0.0%; 0.06%); *p* = 0.001]. Three patients died after TAVI without evidence of an arrhythmic cause according to the available ICM recordings.

**Conclusions:**

Rhythm monitoring for 12 months after TAVI revealed new arrhythmias, mainly AF, in almost one third of patients. Atrial fibrillation burden was higher in patients with prevalent compared to incident AF. Selected patients may benefit from short-term remote monitoring.

**Trial Registration:**

https://clinicaltrials.gov/: NCT02559011.

## Introduction

Little is known about the frequency and burden of conduction disturbances and arrhythmias directly associated with degenerative aortic valve disease. Transcatheter aortic valve implantation (TAVI) for the treatment of severe symptomatic aortic valve stenosis has been established as the treatment of choice among inoperable (1), as well as high (2–4) and intermediate risk patients (5–8) and is a valid alternative for older patients at low surgical risk (9–12). Patients with aortic valve stenosis have a high incidence of both brady- and tachyarrhythmias before and after successful valve replacement. These arrhythmias include atrial fibrillation (AF), atrioventricular (AV) conduction disorder, sick sinus syndrome, ventricular and supraventricular tachycardia and are associated with significant morbidity and mortality. The reported prevalence of pre-existing AF in patients undergoing TAVI ranges from 16 to 50% in various studies (13, 14). New-onset AF (NOAF) after TAVI has been reported in 14–18% of patients after 1 year and 25% after 2 years (13, 15). The incidence of bradyarrhythmia after discharge from TAVI is generally low (4% after 1 year) (15), but may reach 20% in patients with new-onset left bundle branch block (LBBB), including advanced atrio-ventricular (AV) block in 15% of patients, when assessed using an implantable cardiac monitor (ICM) (16). In contrast, sustained ventricular tachycardia is very rare (16). Timely diagnosis and initiation of appropriate therapy may prevent untoward sequelae of arrhythmias, like ischemic stroke, syncope or sudden cardiac death. The purpose of the present study was to investigate the incidence of brady- and tachyarrhythmias among patients with severe, symptomatic aortic stenosis before, during and after TAVI using a small ICM.

## Methods

### Study Population

In this single-center, prospective cohort study, we included 100 patients aged >18 years undergoing TAVI for the treatment of severe symptomatic aortic valve stenosis between March 2016 and October 2019. Patient inclusion was independent of the implanted valve type, access site or baseline heart rhythm. TAVI patients participating in randomized controlled trials ongoing during the same period, patients with a previously implanted permanent pacemaker (PPM) or internal cardioverter defibrillator, patients with clinical contraindications for ICM implantation, and patients unable to give informed consent were excluded from participation in the study. The study was approved by the local ethics committee (KEK-Number 281/15). All study procedures were conducted in accordance with the Declaration of Helsinki. All patients provided written informed consent to participate in the study.

### Study Procedures

All patients included in the study received an ICM (Reveal LINQ^TM^, Medtronic, MN, USA) with remote monitoring capability via the Medtronic CareLink System^TM^ (Medtronic, MN, USA). Whenever possible, we implanted the ICM during pre-TAVI work-up, with the aim to screen for baseline arrhythmias. Implantation was performed subcutaneously according to standard practice in a manufacture-recommended location, as reported elsewhere (17). Details on the programming of the ICMs can be found in the Supplementary Table. All patients received detailed instructions on how to perform remote data transmission and were asked to perform a manual transmission once weekly, in addition to automatic, daily remote transmissions. Staff exclusively responsible for remote monitoring of patients at our institution and well-trained in electrocardiogram analysis triaged all episodes transmitted by the ICMs of study participants. Later during the study, triaging of episodes was provided by FocusOn^TM^ (Medtronic, MN, USA), a specialized triaging service for remote monitoring data. An experienced electrophysiologist adjudicated all triaged electrocardiograms with arrhythmias or unclear findings. If the diagnosis was ambiguous, a second and a third electrophysiologist were consulted and a consensus reached. Data on AF burden was retrieved as displayed in the Medtronic CareLink System.

A 12-lead ECG was recorded in all patients before TAVI, immediately after TAVI and daily thereafter until stabilization of AV conduction or permanent pacemaker (PPM) implantation. Indications for PPM implantation after TAVI were based on institutional and international recommendations (18). Study follow-up included in-office visits or phone calls at 30 days, 3 and 12 months after TAVI. At each time point, a 12-lead ECG was obtained and analyzed according to established recommendations (19). Remote monitoring of the ICM was continuously performed up to study end. If remote transmission failed for longer than 2 weeks, the patients were contacted and remote transmission issues resolved.

### Outcomes

The main study outcome was the diagnosis of new onset arrhythmia within 1-year follow-up after discharge from TAVI and included: AF; sustained supraventricular tachycardia; sustained ventricular tachycardia; advanced AV block; sinus arrest with a pause ≥6 s duration; AF with a pause ≥6 s duration; and bradycardia ≤30 beats per minute for more than 30 s. 12-lead ECGs before TAVI, immediately after TAVI, on day 7 after TAVI or hospital discharge (whichever came first) and after 3 and 12 months after TAVI were analyzed according to established recommendations (19).

### Statistical Analysis

Continuous variables are expressed as means with standard deviations or medians with interquartile ranges (IQR), and categorical variables as frequencies and percentages. Continuous variables were compared using the Mann–Whitney *U*-test or *t*-test while differences in proportions were tested with Pearson's χ2 test or Fisher's exact test. Predictors for AF diagnosis by the ICM were assessed in uni- and multi-variable analyses. Variables with a *p*-value of <0.2 in the crude comparison were selected for adjustment and variable selection in the multiple generalized linear model. Results for survival free from AF or any new arrhythmia with time-to-event data were displayed as Kaplan-Meier curves for descriptive purposes. All tests were performed at a two-sided 5% significance level with two-sided 95% confidence intervals (CIs). All analyses were performed using Stata (StataCorp. Stata Statistical Software: Release 16. College Station, TX: StataCorp LLC).

## Results

**Figure 1:**
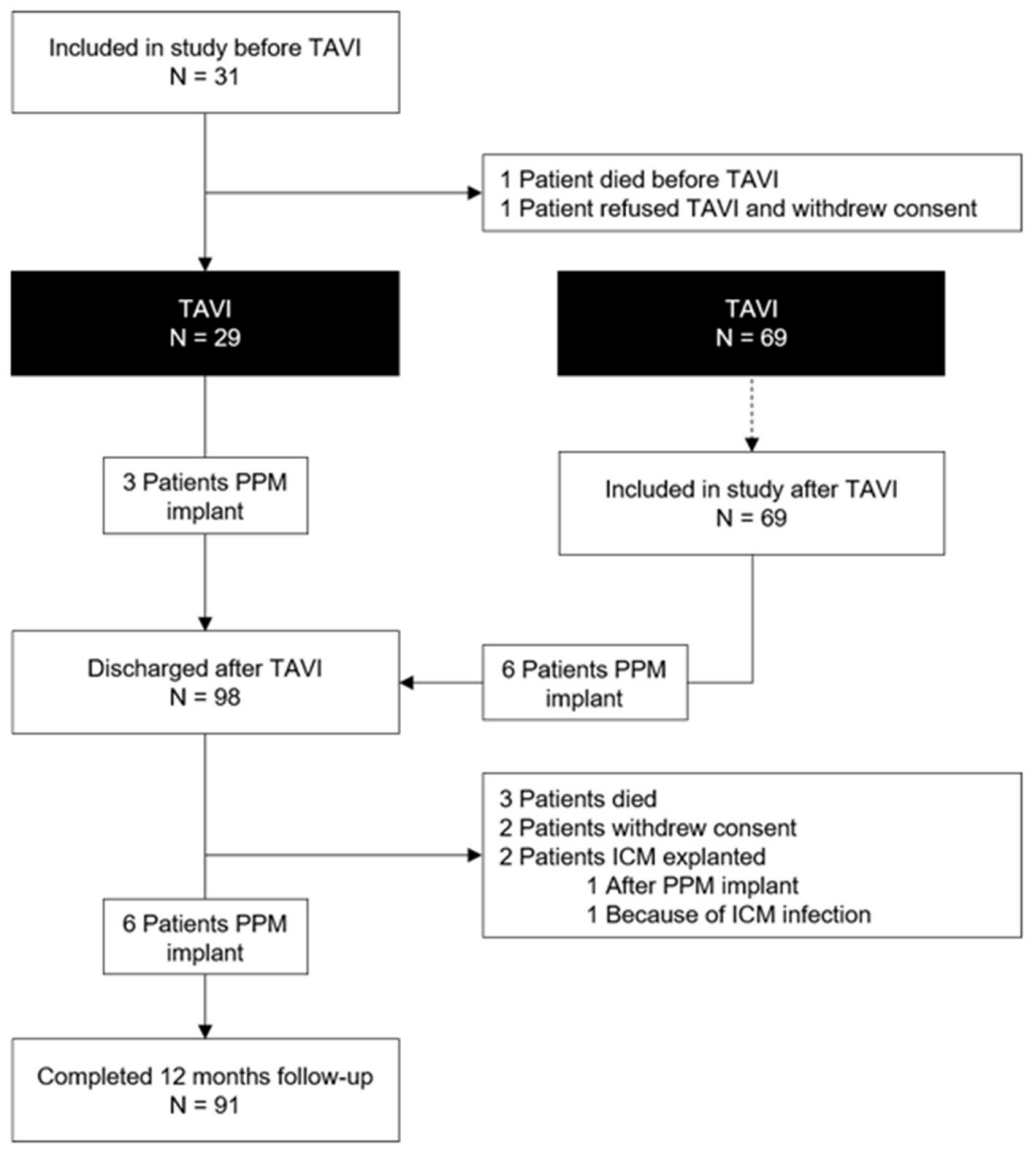
Study flow chart.

Among the 100 study patients, 31 received the ICM a median of 20 days (IQR 4; 29) before TAVI (Figure 1). In the remaining 69 patients, the ICM was inserted a median of 1 day (IQR 0; 2) after TAVI. Table 1 shows the baseline patient characteristics. Four patients died during follow-up, three patients withdrew consent and two patients had their ICM explanted before the end of the study: one because of ICM infection 245 days after TAVI and one after PPM implantation 256 days after TAVI (Figure 1). TAVI was not performed in two patients who had received an ICM (one died prior to the procedure and one withdrew consent for both TAVI and participation in the study).

**Table 1:** Clinical and procedural characteristics.

Age, years	81 ± 5
Gender, female	35 (35%)
Arterial hypertension	89 (89%)
Diabetes mellitus	23 (23%)
Dyslipidemia	71 (71%)
Coronary artery disease	60 (60%)
Peripheral artery disease	9 (9%)
Congestive heart failure	13 (13%)
History of stroke/TIA	10 (10%)
STS Score	3.4 ± 2.1
Atrial fibrillation	28 (28%)
Paroxysmal	16 (57%)
Time since atrial fibrillation diagnosis, months	24 (3; 52)
**Baseline treatment**	
Antiplatelet therapy	99 (99%)
Oral Anticoagulation	32 (32%)
Beta-blockers	55 (55%)
Calcium channel blockers (non-dihydropyridine type)	2 (2%)
Amiodarone	4 (4%)
**Echocardiography**
LVEF, %	59 ± 10
Mean gradient, mmHg	40 ± 14
Peak aortic valve gradient, mmHg	66 ± 22
Aortic valve area, cm^2^	0.7 ± 0.3
Indexed Aortic Valve Area, cm^2^/m^2^	0.2 ± 0.1
Left atrial volume index, ml/m^2^	42 ± 14
TAVI procedure performed	98 (98%)
**Access site (*n*, %)**
Right femoral artery	88 (90%)
Left femoral artery	10 (10%)
**Type of valve (*n*, %)**
Self-expanding valves	41 (42%)
Balloon-expandable valves	52 (53%)
Mechanically-expandable valves	5 (5%)

*Data are provided as mean ± standard deviation, as median with interquartile range (1st; 3rd) or frequencies with percentages. LVEF, left ventricular ejection fraction; STS, society of thoracic surgeons; TAVI, transcatheter aortic valve implantation; TIA, transient ischemic attack*.

**Figure 2:**
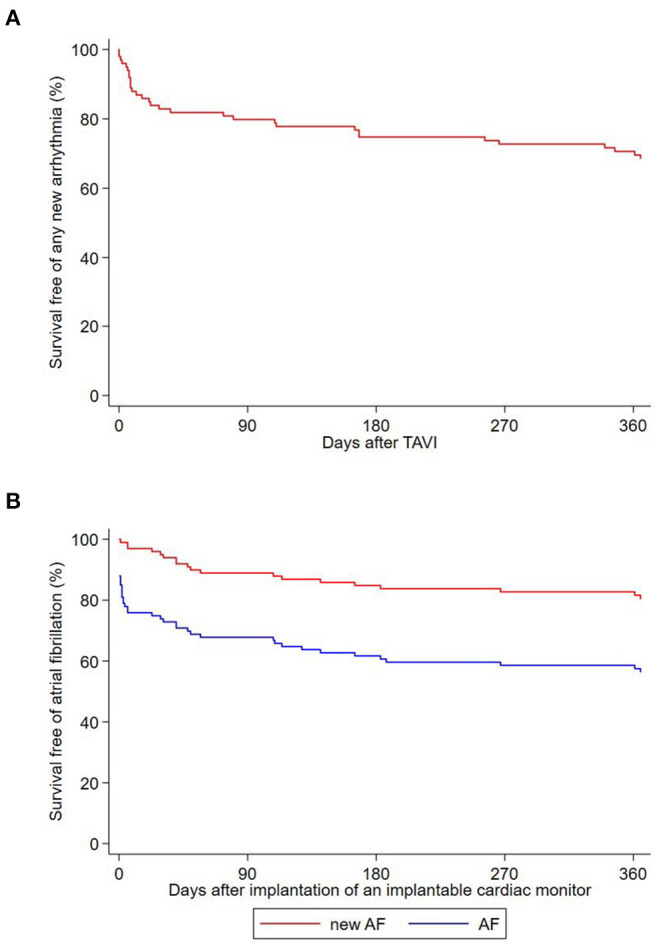
**(A)** Kaplan-Meier survival curve free of any new arrhythmia recorded by the ICM after TAVI. **(B)** Kaplan-Meier survival curve free of atrial fibrillation and new-onset atrial fibrillation after implantation of an implantable cardiac monitor.

The prevalence of baseline right bundle branch (RBBB) and LBBB block was 7 and 10%, respectively. The prevalence of LBBB increased to 39% after TAVI and decreased to 22% after 1 year (Table 2). A PPM was implanted in 15 of the 98 patients who underwent TAVI (15%; Table 3; Figure 1). Nine PPM were implanted before hospital discharge and six thereafter. No patient received a PPM prior to TAVI. Within 1 year after TAVI, a new arrhythmia was observed in 31 patients (31%; Figure 2A). The new arrhythmias were as follows: AF in 19 patients (19%), advanced AV block in three patients (3%), and sustained supraventricular and ventricular tachycardia in 10 (10%) and two patients (2%), respectively.

**Table 2:** ECG characteristics before TAVI, after TAVI, and during follow-up.

**ECG**	**Before**	**Day 1**	Discharge^*^	**3**	**12**
	**TAVI**			**months**	**months**
Number of patients	93 (95%)	94 (96%)	88 (90%)	89 (91%)	86 (88%)
Atrial fibrillation	14 (15%)	16 (17%)	16 (18%)	13 (15%)	13 (15%)
Higher-degree AV block	–	4 (4%)	1 (1%)	1 (1%)	–
PR interval, ms	179 ± 42	185 ± 37	193 ± 43	186 ± 44	187 ± 47
QRS width, ms	106 ± 23	121 ± 28	120 ± 28	109 ± 26	112 ± 28
RBBB	6 (7%)	7 (8%)	4 (5%)	3 (3%)	6 (7%)
LBBB	9 (10%)	34 (36%)	34 (39%)	19 (21%)	19 (22%)
UICD	5 (5%)	4 (4%)	4 (5%)	4 (5%)	5 (6%)

*Data are provided as mean ± standard deviation or frequencies with percentages*.

* *ECG on hospital discharge or day 7, whichever came first*.

*LBBB, left bundle branch block; RBBB, right bundle branch block; TAVI, transcatheter aortic valve implantation; UICD, unspecified, intraventricular conduction disorder*.

**Table 3:** Overview of patients undergoing PPM implantation.

**Case #**	PPM implant^*^	**Days since TAVI**	**Indication of PPM implant**	**ICM finding**	**Device**
11	Before discharge	0	Complete AV block; SSS	No	DDD-PM
22	Before discharge	0	LBBB and PR interval >300 ms	No	VVI-PM
23	Before discharge	2	Complete AV block	No	VVI-PM
29	Before discharge	5	LBBB, AF with pauses >3 s	No	VVI-PM
35	Before discharge	2	LBBB, 2° AV block	No	VVI-PM
40	Before discharge	2	Complete AV block	Yes	DDD-PM
42	Before discharge	0	Complete AV block	No	DDD-PM
43	Before discharge	2	LBBB and increasing PR interval	No	DDD-PM
45	After discharge	10	Complete AV block	Yes	DDD-PM
50	Before discharge	1	Complete AV block	Yes	DDD-PM
51	After discharge	14	Complete AV block	Yes	DDD-PM
53	After discharge	35	SSS	No	VVI-PM
60	After discharge	231	SSS	No	VVI-PM
64	After discharge	9	Complete AV block	Yes	DDD-PM
72	After discharge	36	SSS	No	DDD-PM

* *All PPM were implanted after TAVI*.

*AF, atrial fibrillation; AV, atrioventricular; ICM, implantable cardiac monitor; LBBB, left bundle branch block; PPM, permanent pacemaker; SSS, sick sinus syndrome; TAVI, transcatheter aortic valve implantation*.

*The symbol # indicates number*.

### Atrial Fibrillation

Among the 28 patients with a history of AF at inclusion, 22 were asymptomatic (79%), and AF was paroxysmal in 16 (57%). Overall, AF was recorded by the ICM in 43 patients (43%). The diagnosis of NOAF was made in 19 patients (19%) and in 24 patients (24%) AF was pre-existing (Figure 2). Patients with NOAF initially all had paroxysmal AF. One of these patients developed persistent AF within 12 months after TAVI. Oral anticoagulation was initiated in all after NOAF diagnosis. No AF was observed in four out of the 28 patients (14%) with pre-existing AF. Median time of ICM implantation to first AF recording was 6 days (IQR 0; 93) overall, 57 days (IQR 36; 153) in patients with NOAF, and 1 day (IQR 0; 2) in patients with pre-existing AF (Supplementary Figure). Among the 31 patients with ICM implantation before TAVI, AF was recorded before the procedure in nine patients (29%) at a median of 19 days before TAVI (range 1–96 days before TAVI). AF was pre-existing in seven of these patients (78%) and new in two (22%). AF burden recorded by the ICM in patients with pre-existing AF was 26.7% (IQR 0.3%; 100%), and 0.0% (IQR 0.0%; 0.06%) in patients with NOAF (*p* for difference = 0.001). In patients with pre-existing AF, we found no difference in AF burden before vs. after TAVI [0.0% (IQR 0.0%; 4.8%) vs. 0.1% (IQR 0.0%; 6.1%); *p* = 0.837]. History of stroke or transient ischemic attack, prolonged PR interval or filtered *P* wave duration and larger left atrial volume index (LAVI) were predictors of pre-existing or new-onset AF. In multivariate analysis, larger LAVI and lower mean aortic valve gradient remained significant predictors for AF. Tables 4, 5 show uni- and multivariate predictors for prevalence and incidence of AF.

**Table 4:** Univariate predictors of atrial fibrillation.

	**No AF**	**AF**	*P*-value^*^	**Pre-existing AF**	**New-onset AF**	***P*-value#**
	***N* = 53**	***N* = 47**		***N* = 28**	***N* = 19**	
Age, years	80 ± 5	82 ± 5	0.054	81 ± 5	83 ± 5	0.039
Gender, female	22 (42%)	13 (28%)	0.207	6 (21%)	7 (37%)	0.790
Arterial hypertension	45 (85%)	44 (94%)	0.210	26 (93%)	18 (95%)	0.429
Diabetes mellitus	12 (23%)	11 (23%)	1.000	6 (21%)	5 (26%)	0.759
Dyslipidemia	39 (74%)	32 (68%)	0.660	18 (64%)	14 (74%)	1.000
Coronary artery disease	33 (62%)	27 (57%)	0.685	14 (50%)	13 (68%)	0.783
Peripheral artery disease	5 (9%)	4 (9%)	1.000	2 (7%)	2 (11%)	1.000
Congestive heart failure	8 (15%)	5 (11%)	0.564	4 (14%)	1 (5%)	0.429
History of stroke/TIA	2 (4%)	8 (17%)	0.043	6 (21%)	2 (11%)	0.283
BMI, kg/m^2^	27 ± 5	28 ± 5	0.292	29 ± 5	27 ± 5	0.942
**Electrocardiogram**
PR interval, ms	180 ± 41	205 ± 42	0.007	223 ± 36	193 ± 43	0.235
QRS width, ms	101 ± 23	109 ± 30	0.130	117 ± 32	98 ± 23	0.596
LBBB	12 (23%)	12 (26%)	0.816	8 (29%)	4 (22%)	1.000
RBBB	–	1 (2%)	0.469	–	1 (6%)	0.257
fPWD, ms	146 ± 17	164 ± 23	<0.001	178 ± 25	156 ± 16	0.028
*P* wave integral, μVs	777 ± 263	665 ± 254	0.068	598 ± 247	706 ± 256	0.328
PACS per hour, number	29 ± 71	46 ± 111	0.384	69 ± 147	21 ± 40	0.658
**Echocardiography**
LVEF, %	58 ± 11	59 ± 9	0.927	57 ± 9	61 ± 8	0.451
Mean gradient, mmHg	42 ± 16	38 ± 11	0.139	36 ± 11	41 ± 10	0.737
Aortic valve area, cm^2^	0.6 ± 0.2	0.8 ± 0.3	0.001	0.8 ± 0.3	0.8 ± 0.3	0.003
LAVI, ml/m^2^	37 ± 13	47 ± 13	<0.001	48 ± 13	47 ± 13	0.008
**Laboratory**
BNP, pg/mL	331 ± 622	285 ± 228	0.638	334 ± 245	211 ± 183	0.423
hsTT, μg/L	0.1 ± 0.3	0.1 ± 0.1	0.478	0.1 ± 0.1	0.0 ± 0.0	0.523
hsCRP, mg/L	5.8 ± 6.7	7.8 ± 7.1	0.162	9.2 ± 7.3	5.7 ± 6.3	0.929
Creatinine, μmol/L	122 ± 149	115 ± 83	0.764	122 ± 104	106 ± 35	0.627

*Data are provided as mean ± standard deviation or frequencies with percentages*.

* *Comparison of patients with AF (pre-existing and new-onset) vs. patients without AF*.

*# Comparison of patients with new-onset AF vs. patients without AF*.

*AF, atrial fibrillation; BNP, brain natriuretic peptide; fPWD, filtered P wave duration; hsCRP, high sensitive C-reactive protein; hsTT, high sensitive Troponin T; LAVI, left atrial volume index; LBBB, left bundle branch block; LVEF, left ventricular ejection fraction; PACS, premature atrial contractions; RBBB, right bundle branch block; TIA, transient ischemic attack*.

**Table 5:** Multivariate predictors of atrial fibrillation.

	**Coefficient (95%-CI)**	**OR (95%-CI)**	***p*-value**
**Patients with AF (pre-existing and new-onset) vs. patients without AF**			
Age, years	0.08 (−0.03 to 0.19)	1.08 (0.97–1.21)	0.177
History of stroke/TIA	−0.98 (−2.70 to 0.74)	0.38 (0.07–2.10)	0.264
QRS duration, ms	−0.00 (−0.02 to 0.02)	1.00 (0.98–1.02)	0.920
Mean gradient, mmHg	−0.04 (−0.07 to 0.00)	0.96 (0.93–1.00)	0.053
LAVI (left atrial volume index), mL/m^2^	0.07 (0.03–0.11)	1.07 (1.03–1.11)	0.001
High sensitive C-reactive protein, mg/L	0.04 (−0.04 to 0.12)	1.04 (0.96–1.12)	0.319
**Patients with new-onset AF vs. patients without AF**			
Age, years	0.11 (−0.05 to 0.28)	1.12 (0.95–1.32)	0.164
Filtered P wave duration, ms	0.01 (−0.04 to 0.05)	1.01 (0.97–1.05)	0.696
Aortic valve area, cm^2^	4.06 (0.59–7.52)	57.72 (1.80–1,848.62)	0.022
LAVI (left atrial volume index), mL/m^2^	0.06 (0.01–0.12)	1.07 (1.01–1.13)	0.029

*AF, atrial fibrillation; CI, confidence interval; hsCRP, high sensitive C-reactive protein; fPWD, filtered P wave duration; LAVI, left atrial volume index; OR, odds ratio; TIA, transient ischemic attack*.

### Bradycardia and PPM Implantation

Sinus bradycardia and/or sinus arrest were not observed by the ICM in any patient. AF with a pause lasting 8 s at night was recorded in one patient. No pacemaker was implanted in this patient because he was bedridden. Asymptomatic complete AV block was recorded in five patients with pauses lasting from 2 to 8 s. Complete AV block occurred before discharge from TAVI in two patients and after discharge from TAVI in three patients (6, 7, and 8 days after TAVI). A PPM was implanted in all five patients (Table 3). Second degree AV block type Wenckebach was recorded by the ICM in two additional patients 167 and 300 days after TAVI, respectively. No PPM was implanted in these patients. In three patients, a PPM was implanted during follow-up because of a clinical diagnosis of sick sinus syndrome (symptomatic bradycardia or chronotropic incompetence) 35, 36, and 231 days after TAVI. These patients did not meet bradycardia endpoint criteria defined for the ICM.

### Sustained Ventricular and Supraventricular Tachycardia

Sustained ventricular tachycardias were observed in two patients (2%). One patient had three episodes of asymptomatic ventricular tachycardias lasting from 1 to 8 min. His betablocker dose was increased but he refused further therapies. Another patient had asymptomatic ventricular tachycardia lasting 1 min. After a detailed work-up showing normal left ventricular function, it was decided to continue remote monitoring of the patient without additional interventions. Ten patients (10%) had a median of one sustained supraventricular tachycardia (range 1–6) lasting from 30 s to 1 h and 12 min. All patients with a supraventricular tachycardia were asymptomatic.

### Clinical Outcomes

Table 6 shows the outcomes 30 days and 1 year after TAVI. We were able to retrieve the ICM of the three patients who died after TAVI. The recordings of the ICMs showed either artifacts or asystole, recorded after death in all three patients. No death was causally related to a recorded arrhythmia in any patient. The ICM of the patient who died before TAVI had his last transmission 2 days before death, it showed an increase in heart rate and AF burden. This patient died due to hepatocellular carcinoma and his ICM was not retrievable.

**Table 6:** Clinical outcomes.

**30 days**
All-cause mortality	–
Cardiovascular mortality	–
Any stroke	1 (1%)
Major or life-threatening bleeding	7 (7%)
**1 year**
All-cause mortality	3 (3%)
Cardiovascular mortality	–
Any stroke	1 (1%)
Major or life-threatening bleeding	10 (10%)
Structural valve deterioration	1 (1%)

## Discussion

Rhythm monitoring for 1 year with an ICM in patients undergoing TAVI reveals the following arrhythmias: (1) New-onset atrial fibrillation in one quarter of patients without pre-existing atrial fibrillation; (2) sustained supraventricular tachycardia in one tenth of patients; (3) complete AV block after discharge from TAVI in 3% of patients; and (4) sustained ventricular tachycardia in 2% of patients.

In the MARE multicentric study, 103 patients received an ICM within 3–6 days after TAVI and were followed up during 12 months for relevant arrhythmias (16). In contrast to our study, all patients in the MARE study had new-onset, complete LBBB at inclusion and all patients were included after TAVI. The prevalence of pre-existing AF was 26% compared to 28% in our study. We found an incidence of NOAF of 26% compared to 17% in the MARE study. Using continuous PPM monitoring after TAVI, other authors reported a similar incidence of NOAF of 25% within 1 year (20), while it was 14% 1 year after TAVI in a pooled analysis performed by our group (15). Because the incidence of AF depends on both the screening strategy and the patient population included, higher detection rates are expected in studies using continuous monitoring (21).

Both pre-existing AF and NOAF have been associated with higher mortality and stroke incidence after TAVI (14, 15). Screening for AF and initiation of the appropriate treatment may therefore improve outcome, while predictors of an increased risk of AF are not well investigated in this population. Our prospective study provides some answers and identifies larger LAVI and longer P wave duration as predictors of NOAF, reflecting atrial mechanical and electrical remodeling. Both LAVI and P wave duration are established predictors of AF in other populations and another group also identified left atrial size as the best predictor for NOAF in TAVI patients (22).

Despite high rates of pre-existing AF or NOAF in almost half of TAVI patients, the GALILEO trial, which investigated oral anticoagulation with Rivaroxaban at a dose of 10 mg daily after TAVI compared to antiplatelet therapy in patients without indication for oral anticoagulation, was terminated prematurely because of safety concerns (23). Anticoagulation should therefore only be initiated after unequivocal diagnosis of AF. We observed a significantly lower AF burden in patients with new-onset AF compared to patients with pre-existing AF. There is ongoing debate about the threshold of AF burden that justifies oral anticoagulation, when AF is diagnosed by continuous monitoring (24). The recently published Loop trial failed to show a benefit of oral anticoagulation in patients with screen-detected AF. Randomized trials are ongoing that will shed light onto the threshold of AF burden that justifies oral anticoagulation (25, 26).

We observed complete AV block with consecutive PPM implantation in only three patients after discharge, all within 1 week. A PPM was implanted due to clinical sick-sinus-syndrome in an additional 3% of patients after discharge amounting to a total of 6% of PPM implantation after discharge. In a larger TAVI population, we reported a similar incidence of 6% late PPM implantation after TAVI, of which 16% were due to sick-sinus-syndrome (27). In the MARE study, 10% of patients experienced severe bradycardia leading to PPM implantation, due to either advanced AV conduction impairment or sick sinus syndrome. The higher incidence in the MARE study is most likely the consequence of including a population at higher risk of conduction disturbances (new-onset LBBB after TAVI was present in all), while LBBB at discharge was only present in 39% of the patients in our study. Of note, the prevalence of LBBB decreased by over one third after 12 months, both in the MARE and in our study.

Sustained ventricular tachycardia was rare both in the MARE and the present study. No ICD was implanted in two patients experiencing asymptomatic sustained ventricular tachycardia in our study, whereas two patients received an ICD in the MARE study owing to ventricular arrhythmias. In a larger TAVI population, we have previously described a very low rate of ICD implantation after TAVI (27). With a prevalence of 10%, sustained supraventricular tachycardia were among the most frequent arrhythmias after TAVI. However, they usually lasted only a few minutes, were asymptomatic in all patients and did not require any treatment modifications in our study.

The overall mortality and stroke rates at 12 months (3 and 1%, respectively) were rather low in this elderly population with a mean STS score of 3.4 ± 2.1%. Unfortunately, the functionality of the ICM does not allow accurate conclusions concerning the heart rhythm at the time of death, since bradyarrhythmia and sinus arrest are overwritten by subsequent asystole. Therefore, the only reliable conclusion that can be drawn concerning the occurrence of ventricular arrhythmia, is that they weren't the cause of death in any of the patients.

## Limitations

The present study represents a single center experience at a tertiary care center with follow-up limited to 12 months. Transient complete AV block with a pause of <3 s or 2:1 AV block is not recorded by the ICM, and the prevalence of advanced AV block after TAVI may therefore be underestimated.

## Conclusions

New arrhythmias, mainly AF, were frequent in our TAVI population. Atrial fibrillation burden was higher in TAVI patients with pre-existing AF compared to patients with new-onset AF. The incidence of advanced AV block and of ventricular tachycardia was low.

## Data Availability Statement

The data presented in this article is not readily available because included patients did not consent to share the data with our groups.

## Ethics Statement

The studies involving human participants were reviewed and approved by Kantonale Ethikkommission Bern. The patients/participants provided their written informed consent to participate in this study.

## Author Contributions

All authors listed have made a substantial, direct, and intellectual contribution to the work and approved it for publication.

## Funding

This work was supported by a grant of the Swiss National Foundation (32003B_163059) to SW. Medtronic (Minneapolis, US) provided the ICMs (REVEAL LinQ^TM^) free of charge. Medtronic was not involved in the study design, collection, analysis, interpretation of data, the writing of this article or the decision to submit it for publication.

## Conflict of Interest

The spouse of JS is an employee of Boston Scientific. HT reports educational grants from Biosense Webster and travel grants from Abbott. FN reports travel fees from Medtronic, Abbott, Boston Scientific and Philips Spectranetics, speaker fees from Medtronic and Abbott, educational grants from Medtronic, Abbott, Boston Scientific, Philips Spectranetics and Actinno and institutional grants from Biotronik. AH received travel/educational grants from Medtronic and Philips/Spectranetics. He is consultant/advisor for DiNAQOR and Biotronik and Co-founder/head of Act-Inno. SS reports research grants to the institution from Edwards Lifesciences, Medtronic, Boston Scientific and Abbott, as well as personal fees from Boston Scientific, Teleflex and BTG. TP reports research grants to the institution from Biotronik, Boston Scientific and Edwards Lifesciences; speaker fees from Biotronik and Boston Scientific; Clinical event committee for study sponsored by HighLifeSAS; travel reimbursement from Medira; proctoring for Medtronic. SW reports research and educational grants to the institution from Abbott, Amgen, Astra Zeneca, BMS, Bayer, Biotronik, Boston Scientific, Cardinal Health, CardioValve, CSL Behring, Daiichi Sankyo, Edwards Lifesciences, Guerbet, InfraRedx, Johnson & Johnson, Medicure, Medtronic, Novartis, Polares, OrPha Suisse, Pfizer, Regeneron, Sanofi-Aventis, Sinomed, Terumo, V-Wave. He serves as unpaid advisory board member and/or unpaid member of the steering/executive group of trials funded by Abbott, Abiomed, Amgen, Astra Zeneca, Bayer, BMS, Boston Scientific, Biotronik, Cardiovalve, Edwards Lifesciences, MedAlliance, Medtronic, Novartis, Polares, Sinomed, Terumo, V-Wave and Xeltis, but has not received personal payments by pharmaceutical companies or device manufacturers. He is also member of the steering/executive committee group of several investigator-initiated trials that receive funding by industry without impact on his personal remuneration. TR reported research grants from the Goldschmidt-Jacobson Foundation, the Swiss National Science Foundation, the Swiss Heart Foundation and the sitem-insel Support Funds, all for work outside the submitted study; advisory board membership, speaker and travel support from Abbott/SJM, Astra Zeneca, Brahms, Bayer, Biosense-Webster, Biotronik, Boston-Scientific, Daiichi Sankyo, Medtronic, Pfizer-BMS and Roche, all for work outside the submitted study and without impact on his personal remuneration; as well as support for his institution's fellowship program from Abbott/SJM, Biosense-Webster, Biotronik, Boston-Scientific and Medtronic for work outside the submitted study and without impact on his personal remuneration. FP reports travel expenses from Abbott Vascular, Edwards Lifesciences, and Polares Medical. LR received speaker honoraria from Abbott/SJM and consulting honoraria from Medtronic. The remaining authors declare that the research was conducted in the absence of any commercial or financial relationships that could be construed as a potential conflict of interest.

## Publisher's Note

All claims expressed in this article are solely those of the authors and do not necessarily represent those of their affiliated organizations, or those of the publisher, the editors and the reviewers. Any product that may be evaluated in this article, or claim that may be made by its manufacturer, is not guaranteed or endorsed by the publisher.

## Abbreviations

AF: Atrial fibrillation
AV: Atrioventricular
ICM: Implantable cardiac monitor
LAVI: Left atrial volume index
LBBB: Left bundle branch block
NOAF: New-onset atrial fibrillation
PPM: Permanent pacemaker
RBBB: Right bundle branch block
TAVI: Transcatheter aortic valve implantation.
