# Supplementary material for: Assessment of New Onset Arrhythmias After Transcatheter Aortic Valve Implantation Using an Implantable Cardiac Monitor
Source: Front Cardiovasc Med. 2022 May 16;9:876546. doi: 10.3389/fcvm.2022.876546 (PMC9149277; doi:10.3389/fcvm.2022.876546)
Supplement: Supplementary file 1 [file Data_Sheet_1.docx]

**Supplementary Material**

Supplementary Table. Programming of ICM.

| **Parameter** | **Setting** |
| --- | --- |
| AT/AF detection | On |
| Type | AF Only |
| AF Detection | Balanced Sensitivity |
| Ectopy rejection | Nominal |
| AT/AF recording threshold | All episodes |
| Reason for monitoring | Syncope |
| Wireless data priority | Pause, tachy, brady |
| Wireless transmission time | 04:00 |
| Tachy | On / 420 ms (143 bpm) / 16 beats |
| Brady | On / 2000 ms (30 bpm) / 4 beats |
| Pause | On |
| AT/AF | AF only |
| Sensitivity | 0.035 mV / 35 μV |
| Blank after sense | 150 ms |
| Sensing threshold deca delay | 150 ms |

**Supplementary Figure**

Time to AF diagnosis after ICM implantation in patients with pre-existing and new-onset AF. AF: atrial fibrillation; ICM: implantable cardiac monitor.
